# Factors controlling bark decomposition and its role in wood decomposition in five tropical tree species

**DOI:** 10.1038/srep34153

**Published:** 2016-10-04

**Authors:** Gbadamassi G. O. Dossa, Ekananda Paudel, Kunfang Cao, Douglas Schaefer, Rhett D. Harrison

**Affiliations:** 1 Key Laboratory of Tropical Forest Ecology, Xishuangbanna Tropical Botanical Garden, Chinese Academy of Sciences, Menglun, Mengla 666303, Yunnan, China; 2 Centre for Mountain Ecosystem Studies, Kunming Institute of Botany, Kunming 650201, Yunnan, China; 3 University of Chinese Academy of Sciences, Beijing 100039, China; 4 Ecophysiology and Evolution Group, State Key Laboratory for Conservation and Utilization of Subtropical Agro-Bioresources, and College of Forestry, Guangxi University, Nanning, Guangxi Province, 530004, China; 5 World Agroforestry Centre, East & Central Asia Regional Office, Kunming 650201, Yunnan, China

## Abstract

Organic matter decomposition represents a vital ecosystem process by which nutrients are made available for plant uptake and is a major flux in the global carbon cycle. Previous studies have investigated decomposition of different plant parts, but few considered bark decomposition or its role in decomposition of wood. However, bark can comprise a large fraction of tree biomass. We used a common litter-bed approach to investigate factors affecting bark decomposition and its role in wood decomposition for five tree species in a secondary seasonal tropical rain forest in SW China. For bark, we implemented a litter bag experiment over 12 mo, using different mesh sizes to investigate effects of litter meso- and macro-fauna. For wood, we compared the decomposition of branches with and without bark over 24 mo. Bark in coarse mesh bags decomposed 1.11–1.76 times faster than bark in fine mesh bags. For wood decomposition, responses to bark removal were species dependent. Three species with slow wood decomposition rates showed significant negative effects of bark-removal, but there was no significant effect in the other two species. Future research should also separately examine bark and wood decomposition, and consider bark-removal experiments to better understand roles of bark in wood decomposition.

Ecosystem processes depend in part on decomposition of organic matter. Globally, litter and soil decomposition produces more CO_2_ than fossil-fuel combustion^1^. It is also the most important pathway for returning nutrients to soils for plant uptake. Many studies focus on factors affecting decomposition of plant parts including leaves, wood and roots. However, bark decomposition has received comparatively little attention^2^. Nonetheless, bark decomposition is interesting because: (i) bark can comprise up to 20% of the dry mass of tree stems, (ii) bark forms a substantial component of litter in some forests; some species (*e.g., Eucalyptus*) shed bark as they grow, and in harvesting wood, logs are often stripped of their bark in the field, and (iii) bark can potentially affect the rate of wood decomposition through altering decomposer access^3^, and the microclimate and chemical conditions within decaying logs. The importance of bark for wood decomposition is implicit in the fact that log decay classes are based on the presence or absence of bark^4^.

Most existing studies on bark decomposition have been done in European boreal or North American temperate forests, with to our knowledge only one recent study in the tropics^5^, although bark is much more variable in the tropics^6^. Some studies have indirectly examined decomposition of bark while still attached to wood^7^,^8^,^9^,^10^,^11^ or stumps^10^. Others tested the efficacy of microorganisms in decomposing bark (*e.g.*, white rot fungi^12^,^13^, soil fungi^14^,^15^, or bacteria^16^) or investigated the role of the physical structure of bark by grinding it to powder^14^,^17^. The scarcity of studies on bark decomposition, especially in the tropics, is due in part to the fact that it is often considered an integral part of wood, although it has very different physical and chemical properties. Different authors have suggested that the presence or absence of bark may inhibit or enhance the decomposition of dead wood, but few studies have tested these ideas. For example, the bark of some species contains compounds that are known to inhibit microorganisms (*e.g., Tectona grandis* bark^18^). Physiological and mechanical functions of bark have been investigated in living trees and several distinct functions, including defense against pests and herbivores, reduction of desiccation, protection against fire, control in trunk girth seasonal changes, mechanical support, storage, and photosynthesis, have been attributed to bark^6^,^19^,^20^,^21^,^22^,^23^. However, to what extent these functions, especially the defensive ones, are still important after tree death remains poorly understood^2^. For example, a recent article highlights the potential role of bark traits in influencing faunal assemblages during the early stages of wood decomposition^3^. Arthropods or invertebrate fauna in general are recognized to influence organic matter decomposition not only directly through the amount of matter consumed (mainly by termites in tropical and subtropical environments) but also through indirect effects such as grazing on fungi sporocarps^5^.

Bark is the outer layer beyond the living cambium, which often has corky structure and is composed of dead cells^24^. It can represent up to 20% of the dry weight of the tree stem^13^. Bark and wood differ markedly in chemical composition and structure. Bark is mostly suberin. Among the functions attributed to suberin are desiccation reduction and inhibition of attack from microorganisms^25^. Bark also contains higher nutrient concentrations and C than wood^19^,^26^,^27^. Since chemical composition of litter is the most important factor after climate in determining decomposition rates^28^, one may anticipate that bark and wood will differ substantially in their decomposition rates. Indeed, some studies have already shown that nutrient dynamics during decomposition differ between bark and wood^11^. A recent study also showed that lignin and polyesters such as cutin and suberin responded differently to temperature in a warming experiment^29^. In addition, for logs there is a substrate size – decomposition rate relationship^30^, whereby larger logs decompose more slowly. This relationship may in part be linked to bark effects on decomposition rates, because smaller logs have a larger proportional surface area and hence a higher proportion of bark. Thus, coupling bark decomposition studies with additional experiments investigating the decomposition of logs with and without bark is likely to enhance our understanding of the mechanisms affecting wood decomposition.

We therefore investigated factors affecting bark decomposition and the effects of bark on wood decomposition for five important tree species in a secondary seasonal tropical rain forest in Xishuangbanna, SW China. We employed a common litter-bed approach to minimize environmental variation and thus better understand the effects of species traits on bark decomposition. We examined the following hypotheses: (i) Bark contains higher C and N than wood among the study species, (ii) bark decomposition rates decline across species as the proportions of lignin, cellulose and tannins in bark tissue increase; (iii) meso- and macro-fauna promote bark decomposition, both through enhancing microbial decomposition and fragmentation of bark; and (iv) logs retaining bark decompose faster than logs without bark, and this effect decreases with bark decomposability.

## Results

### Initial characteristics of bark litter and branches

When bark and wood chemistries are compared, in four of the five species (the exception being *Kleinhovia hospita*) wood tissue contained more carbon and cellulose but less N and P than bark tissue (Table 1). *Kleinhovia hospita* had substantially the highest N, P and cellulose contents in both bark and wood, and the wood of *Kleinhovia hospita* contained less cellulose and water-soluble sugar than bark. The patterns for lignin, hemicellulose and K were mixed (Table 1). The tannin content of bark tissue increased in the following order: *Kleinhovia hospita* << *Tectona grandis* *<* *Cunninghamia lanceolata* *<* *Dipterocarpus turbinatus* *<* *Toona ciliata.* Meanwhile, wood specific gravity increased in the following order: *Kleinhovia hospita* *<* *Tectona grandis* *<* *Toona ciliata* *<* *Cunninghamia lanceolata* < *Dipterocarpus turbinatus* (Table 1).

### Nutrient dynamics in bark litter during decomposition

**Figure 1:**
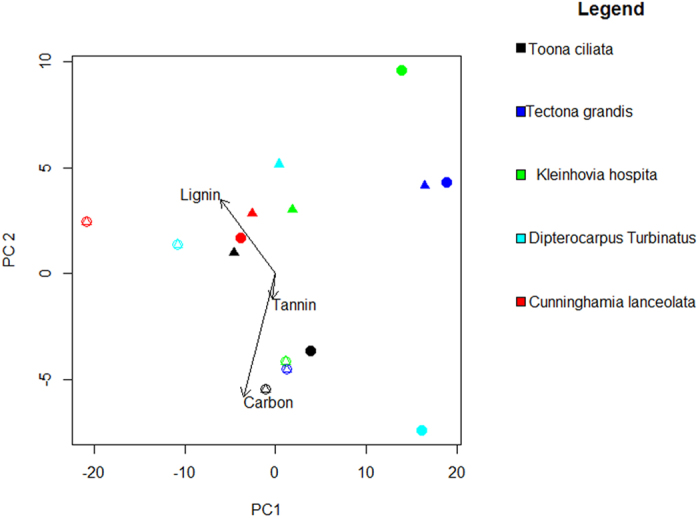
Principal components analysis (PCA) of bark litter from litter-bags at the start (0 mo, open symbols) and end (12 mo, filled) of incubation in a common litter-bed in a secondary rain forest at Xishuangbanna Tropical Botanical Garden for five tree species (*Kleinhovia hospita, Tectona grandis, Cunninghamia lanceolata, Dipterocarpus turbinatus,* and *Toona ciliata*) with respect to faunal exclusion (triangles; fine mesh = 0.068 mm) and faunal access (circles; coarse mesh = 4.75 mm upper side). Values are laboratory duplicate averages. For 12 mo samples, due to the amount of remaining bark, only two samples (n = 2) per bag type and species were obtained for analysis. For details of changes in the concentrations of specific bark substances through time please refer to Fig. S1.

Regardless of the species and litter-bag treatment, sugar, cellulose and carbon concentrations in bark litter decreased between the initial (0 month) and final samples (after 12 months of incubation), whereas nitrogen concentrations increased (Fig. 1, Fig. S1). For other nutrients, no general trends were found. The first two axes of a principal component analysis (PCA) explained 96% of the variation in bark chemistry among species and samples taken at both the start and end of the experiment (PCA1, 82.01% and PCA2, 14.33%). C, N, lignin and tannin were important in explaining the variation bark litter chemistry (Fig. 1). The PCA reveals a strong species effect in the change in bark chemistry through decomposition, but the mesh size effect was species dependent. There were large differences in nutrient concentrations between bark litter remaining at the end of the experiment in coarse mesh and fine mesh bags for three species, but not for *Tectona grandis* and *Cunninghamia lanceolata.*

### Bark litter decomposition

**Figure 2:**
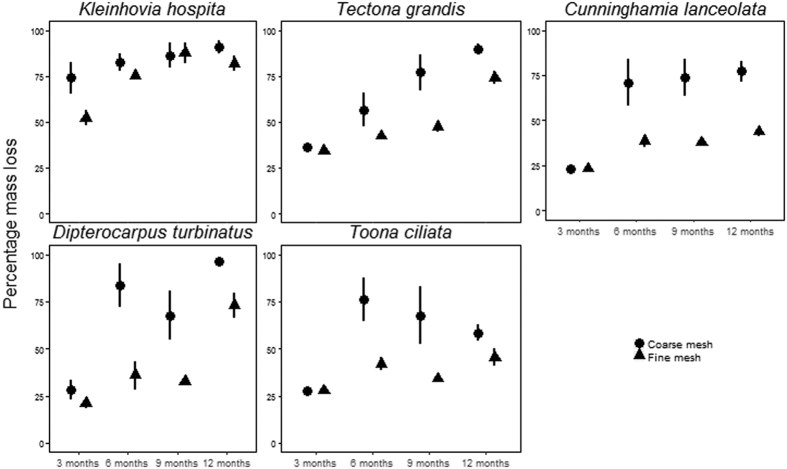
Percent mass loss of bark litter from litter bags after 3 mo, 6 mo, 9 mo and 12 mo incubation in a common litter-bed in a secondary rain forest at Xishuangbanna Tropical Botanical Garden for five tree species (*Kleinhovia hospita, Tectona grandis, Cunninghamia lanceolata, Dipterocarpus turbinatus,* and *Toona ciliata*) with respect to faunal exclusion (fine mesh, mesh size = 0.068 mm) and faunal access (coarse mesh, mesh size = 4.75 mm upper side). Five litter bags per treatment were harvested at 3 mo, 6 mo and 12 mo and 3 bags were harvested at 9 mo. Data represent means ±SE.

The optimal model for bark litter decomposition retained number of days (polynomial 2^nd^ degree), species, litter-bag type and all two-way interactions as independent variables. To correct for heteroscedasticity, the exponential function (varExp (form = ~fitted(.)) was used. After 1 yr incubation, bark litter mass loss ranged from 96.45 ± 0.54% (mean ± standard error, N = 5) for *Dipterocarpus turbinatus* to 58.49 ± 4.15% for *Toona ciliata* in coarse mesh bags and from 81.96 ± 4.39% for *Kleinovia hospita* to 43.78 ± 2.63 for *Cunninghamia lanceolata* in fine mesh bags (Fig. 2). Across all species, bark litter in coarse-mesh bags had higher mass loss than in fine-mesh litter-bags (Table 2). The variance in decomposition rates was much higher for coarse mesh bags than for fine mesh bags. There was a strongly significant species effect (Table 2 and Table S1), a significant mesh size:time interaction (Table 2), and a significant species:time interaction (Table 2). All species had rapid decomposition over the first six months, which was during the wet season. However, *Kleinhovia hospita* decomposed more rapidly over the first three months, compared to other species. Post-hoc pairwise tests for bark decomposition among species revealed three significantly distinct groupings irrespective of litter-bag treatments; decomposition rates increased in the following order: (i) *Cunninghamia lanceoleta* and *Toona ciliata* (ii) *Tectona grandis,* and (iii) *Kleinhovia hospita. Dipterocarpus turbinatus* could not be distinguished from groups (i) and (ii) (Table S2).

### Log decomposition

**Figure 3:**
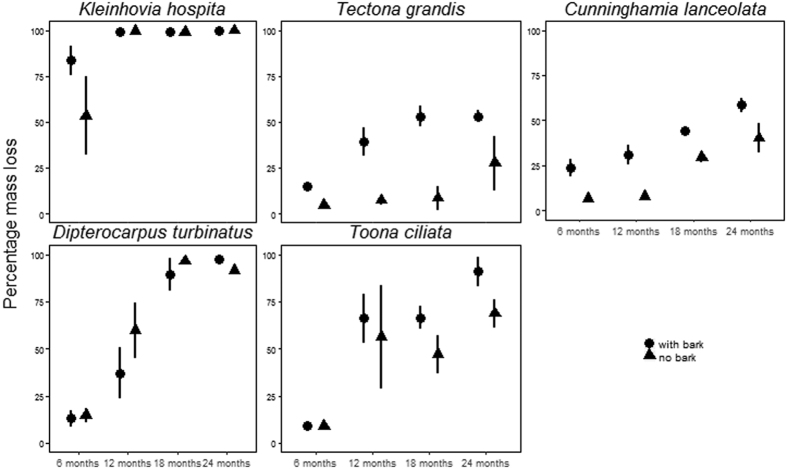
Percent mass loss of branches after 6 mo, 12 mo, 18 mo and 24 mo incubation in a common litter bed in a secondary rain forest at Xishuangbanna Tropical Botanical Garden for five tree species (*Kleinhovia hospita, Tectona grandis, Cunninghamia lanceolata, Dipterocarpus turbinatus,* and *Toona ciliata*) with respect to bark removal treatment (bark intact vs bark removed). Three branches were collected every six months. Data represent means ±SE.

The optimal model of branch mass loss over two years retained number of days (polynomial 2^nd^ degree), species, bark-removal treatment and all two-way interactions as independent variables. To correct for heteroscedasticity, we employed the variance identity (varIdent) function to permit variances among both species and bark treatment to vary. We found that main effect of bark removal was not significant (Table 3, Fig. 3, Table S3). However, there was a significant bark-treatment:species interaction. Logs with bark decomposed faster than those without for *Toona ciliata* (1.33 fold increase), *Cunninghamia lanceolata* (1.46 fold increase) and *Tectona grandis* (1.92 fold increase), but not for *Kleinhovia hospita* and *Dipterocarpus turbinatus* (Fig. 3). As expected, there was also a strong species effect on the rate of branch decomposition and also a significant species:time interaction (Table 3). The species with the fastest decomposition rate was *Kleinhovia hospita.* Regardless of the treatment, *Kleinhovia hospita* completely decomposed within 12 mo. The slowest to decompose was *Tectona grandis* (53.1 ± 5.5% for branches with bark and 27.6 ± 25.70% for branches without bark over 24 mo). Post-hoc pairwise tests for decomposition of branches with bark among species revealed three distinct groupings. Decomposition rates increased in the following order: (i) *Tectona grandis and Cunninghamia lanceolata,* (ii) *Toona ciliata* and *Dipterocarpus turbinatus*, and (iii) *Kleinhovia hospita*. For branches without bark four distinct groups were found. Decomposition rates increased in the following order (i) *Tectona grandis*, and *Cunninghamia lanceolata,* (ii) *Toona ciliata,* (iii) *Dipterocarpus turbinatus,* and (iv) *Kleinhovia hospita* (Table S4).

## Discussion

This experiment examined the decomposition of both bark litter and branches of five important tropical tree species, using a common litter-bed approach. Previous studies have revealed that climate^28^,^31^, litter substrate quality^28^ and decomposer community composition^32^,^33^ are principal drivers of variance in decomposition rates. Through using a common litter bed approach we controlled for environmental effects, including climate and decomposer community composition.

For bark litter, we found that litter in mesh bags that permitted meso- and macro-faunal access (*i.e*. coarse mesh) decomposed faster, regardless of wood species. There was also substantial variation in bark decomposition rates among species. *Kleinhovia hospita* with substantially lower bark tannin content decomposed fastest, especially in fine-mesh bags. However, contrary to our hypothesis differences in decomposition rates among the other four species did not follow variation in tannin content. For bark litter in coarse-mesh bags, there was very high within-species variance in decomposition rates particularly after 6 mo, which appeared to reflect termite activity. For wood decomposition, as predicted branches with bark decomposed faster than without bark in three species and in the remaining two species there were no significant effects of bark removal. The species with significant bark removal effects had the slowest decomposing wood.

Our study is the first to have examined differences in chemistry between bark and wood in tropical tree species. Among temperate species, Harmon *et al*.^34^ and Martin *et al*.^27^ found C content to be higher in bark than wood. In our study, we found the opposite. Likewise, for N content, Martin *et al*.^27^ reported higher concentrations in bark than in wood, which was opposite to the pattern in our study. These differences between our results and those reported previously from temperate species may simply reflect the small number of species examined. However, if the results do reflect differences in tissue-construction strategy between tropical and temperate species, it would suggest a wider sampling of tree species would be rewarding.

The relative increase in the concentration of N through decomposition is perhaps surprising, but can possibly be explained by the high N content of tannins and other secondary metabolites that are resistant to decomposition. The two species for which mesh size did not have any effect on the changes in relative nutrient concentrations were the two species with the most recalcitrant bark litter.

Bark litter in coarse-mesh decomposed faster (1.11–1.76 fold increase) than in fine-mesh bags across all species, indicating an important effect of litter meso and macro invertebrates on decomposition of bark in this environment. In a recent review, Ulyshen^35^ found that arthropods are capable of consuming up to 20% of decomposing wood litter. Colonisation by invertebrates is also likely to facilitate microbial decomposition through the increase in substrate surface area resulting from fragmentation and microbial inoculation. High within-species variation in decomposition rates of bark in coarse-mesh bags could be explained by differences in arthropod colonization patterns, most likely termites. Other bark factors, such as water-holding capacity^5^,^36^, toughness and surface structure of bark tissue, may together affect the attractiveness of bark litter to litter fauna and the rate of microbial decomposition. However, in our experiment there was no significant interactive effect between tree species and mesh size, indicating that specific characteristics of the litter did not alter effects of meso- and macro-faunal access. Nevertheless, there was a strong direct effect of species on bark litter decomposition rates. Recent findings from Zuo *et al*.^3^ highlight that for the early stages of decomposition, bark traits potentially determine the faunal assemblage on logs, which perhaps could explain part of the variation in wood decomposition earlier explained by species identity. Litter quality has long been identified as key controlling factor for the decomposition of organic matter^37^. Decomposition by fungi is generally limited by N availability^37^, which may explain why the bark of *Kleinhovia hospita*, with the highest initial N content, decomposed most rapidly in fine-mesh bags. *Kleinhovia hospita* bark also had the highest cellulose to lignin ratio and microorganisms tend to perform well in presence of high cellulose to lignin ratios^30^. Conversely, the low cellulose to lignin ratio in the bark of *Cunninghamia lanceolata* may explain why it had the slowest decomposition rate in fine-mesh bags. Moreover, *C. lanceolata* is a gymnosperm and as Weedon *et al*.^37^ and Cornwell *et al*.^28^ point out, gymnosperms usually contain lignin made from guaiacyl, which is a highly resistant compound.

As expected, in three species branches with bark decomposed faster than branches without bark. In the other two species bark removal did not have any significant effect. Recently Johnson *et al*.^11^ suggested that variation among species in rates of wood decomposition may be a consequence of differences in bark mineralization. Bark may have a moisture-retention function in woody debris due to the presence of suberin in bark, thereby enhancing microbial decomposition through providing a favorable micro-environment. Ulyshen *et al*.^5^, who studied bark removal in a single species (*Liquidambar styraciflua* L.) also found the presence of bark enhanced wood decomposition rates. Bark may also alter overall substrate quality. It is noteworthy that in our experiment the effect of bark removal was significant in the three species with the slowest decomposing branches: *Tectona grandis, Cunninghamia lanceolata* and *Toona ciliata*. Perhaps, for these species, chemical constituents released from the decomposition of bark tissue facilitate the decomposition of wood, which is otherwise highly recalcitrant.

As expected, we found a significant effect of species on the rate of decomposition of branches. *Kleinhovia hospita*, whose branches were the fastest to decompose regardless of bark treatment, had the lowest wood specific gravity, highest concentration of N, P, and K, and lowest carbon to nitrogen ratio, while its bark had the lowest tannin content and highest cellulose to lignin ratio. *Cunninghamia lanceolata* branches with and without bark were the second slowest to decompose, which may be explained by the slow colonization and establishment of fungi on gymnosperms as compared to angiosperms^28^,^38^. We found a significant species:time interaction in the decomposition of branches, with the slower decomposing species evidencing slower initial decomposition (Fig. 3). These results corroborate those recently reported by Schilling *et al*.^39^ who tracked the decomposition of eight woody species in a tropical dry forest. Moreover, previous studies recognized *Cunninghamia lanceolata* as a highly decay-resistant species and especially resistant to fungal attack^40^, perhaps because of the high lignin content of its bark and wood and also because its heartwood contains antifungal properties^41^. Branches of *Tectona grandis* were the slowest to decompose likely because as reported by several studies this species both wood and bark contain some antifungal properties^42^,^43^,^44^. Our *Tectona grandis* branches with bark decomposed faster than those without bark. Previous research also found that the decay resistance of *Tectona grandis* wood increased with the depth (*i.e.* sapwood decompose faster than heartwood which was correlated with the concentration of antifungal extracts^42^). Moreover, *Tectona grandis* wood itself is also well recognised for its resistance to termite attack^45^.

In this study we did not investigate the effect of bark removal with respect to log diameter, which is likely to be important^21^. In addition to surface area:volume effects, the thickness, physical structure and chemical composition of bark may vary among woody tissues of different size, although Rosell *et al*.^24^ recently showed that bark from branches and twigs was representative of bark on the trunk among 85 species of angiosperms. Our chemical analyses also did not include important (but difficult to measure) bark components, such as lignin monomers or suberin. Improved understanding of the distribution and role of these substances in decomposition may be important to understanding bark decomposition and its role in the decomposition of wood. In addition to repeating our experiments across an expanded sample of species, a comprehensive survey of functional relationships among bark and wood characteristics, and their evolutionary and ecological patterns would be very informative.

In summary, organic matter decomposition, carbon and nutrient cycling are important topics for ecological study. However, recently, the need to further parameterize and provide better mechanistic understanding of decomposition processes, has resulted in calls for more tissue specific approaches. Until recently, researchers have usually regarded wood and bark as a single substrate. Indeed, assignment of wood decay classes has relied mostly on the status of bark (presence/absence, state of decomposition). Here, we investigated bark and wood decomposition in five tropical tree species. We found that species traits and litter fauna access had important effects on bark litter decomposition. Through a bark removal experiment, we also found that bark enhanced the rate of wood decomposition in some species, but had no effect in others. Species with a significant bark removal effect were those with the slowest rates of wood decomposition.

## Materials and Methods

This study was conducted in a secondary rainforest (UTM 47Q, 0734280 E, 2425546 N, 589 m als.) at Xishuangbanna Tropical Botanical Garden (XTBG), a seasonal moist tropical forest. Total annual rainfall is 1463 mm but rainfall is highly seasonal with 87% of total precipitation falling between May and Oct^46^. However, fog during the cool dry season (Nov–Mar) reduces plant water deficits, permitting tropical rain forest to persist^46^. Monthly mean temperature varies from 15.9 °C in Jan to 25.7 °C in June^47^.

We conducted an experiment using litter bags to assess bark decomposition over 1 yr from May 9^th^ 2014 to May 9^th^ 2015. In addition, we monitored the decomposition of 5-cm diameter branches with and without bark over 2 yrs from Nov. 23^rd^ 2013 to Nov. 4^th^ 2015.

### Species selection

We selected tree species which met at least two of the following criteria: (i) important timber producing tree species for the Asian timber-trade market, (ii) species that are ecologically important in Asian tropical forests and (iii) tree species used extensively for reforestation or in silvicultural plantations in Asia. Species were also selected to represent a range of wood densities and supposed decay resistance. Finally, the species also all had to be present at XTBG, so that their wood was available. In total, five species; *Kleinhovia hospita* L. (Malvaceae)*, Tectona grandis* L.f. (Lamiaceae)*, Cunninghamia lanceolata* Hook. (Cupressaceae)*, Dipterocarpus turbinatus* C.F. Gaertn. (Dipterocarpaceae), and *Toona ciliata* M. Roem. (Meliaceae) were selected.

### Bark collection and preparation

Bark was removed from ~5 cm diameter branches of these tree species. Branches were from one tree for *Cunninghamia lanceolata, Toona ciliata*, and *Tectona grandis*, two trees for *Kleinhovia hospita* and from three trees for *Dipterocarpus turbinatus*. The number of trees used was determined by the amount of material produced during the regular pruning operations of the horticultural department at XTBG. The removed bark material was cut into pieces of ca. 1 cm × 5 cm. This was to ensure that bark from different species was presented in more or less same physical shape and also so that it could be fitted into the litter-bags. The collected bark litter was then dried at 80 °C for 24 hrs, frozen at −80 °C for 24 hrs and dried again at 80 °C for 24 hrs to defaunate it^48^,^49^.

### Litter-bags

To study effects of fauna on the decomposition of bark, we used two types of litter-bags. Coarse mesh (mesh size 4.75 mm for upper side, and mesh size 0.5 mm lower side to reduce loss of small particles from fragmentation) bags allow litter fauna access to the bark litter. Our fine mesh (mesh size 0.068 mm) bags excluded all arthropods and mesofauna^32^. Litter-bags were incubated within a 3.6 m × 2.4 m common litter-bed. At 3-mo intervals we harvested litter-bags. Five replicates were harvested at 3, 6 and 12 mo after incubation, and three replicates were harvested at 9 mo.

Each litter-bag was initially filled with 20 g of dry bark litter. Upon collection, each litter-bag was checked visually for termites and then the remaining material in each bag was carefully and gently cleaned with tap water before oven drying at 105 °C to constant mass. Any fine mesh bags with evidence of termite holes were rejected, as in addition to termites access by other meso- and macrofauna may have occurred. Coarse-mesh bags attacked by termites were retained for analysis. The remaining bark litter dry mass was measured to 0.01 g using an electronic balance.

### Branches collection, preparation, incubation and harvest

Fresh branches were cut from the target species while the garden’s horticultural staff were conducting routine pruning operations. Branches were transported to the laboratory, where an electric saw was used to cut branches into pieces of ~0.5 m length. The saw was sterilized with ethanol and flamed between cuts to minimise transfer of microbes, in particular fungi, between samples. Branches with irregular shapes were not used. Each branch was tagged with a uniquely numbered metal tag and both ends were coated with black enamel paint to reduce arthropod colonization into cuts^50^. We recorded the (i) diameter (at both ends and in the middle) as measured using a diameter tape, (ii) bark thickness measured at both ends with a digital caliper, (iii) fresh weight of the entire log, and (iv) the total length. For branches with bark removed, these metrics were recorded before and after bark removal. Disks taken at the base of each branch prior to preparation were stored at −20 °C until chemical analyses.

Three replicates of each species per treatment (with/without bark) were collected at 6, 12, 18 and 24 mo after incubation. If branches became fragmented, we carefully collected each fragment that was visible to naked eye using forceps. After harvesting, we recorded the presence/absence of termites and then mud, sand, insect frass and mosses were carefully brushed off the branch. The fresh weight of each branch was measured to 0.01 g using an electronic balance. Next, three disks of 2 cm thickness were cut from the middle of the branch and approximately 5 cm from each end (to avoid end effects) (Fig. S2). For branches that were fragmented, we used several pieces instead of three disks. Each disk was weighed before and after drying at 105 °C to constant mass. The dry volume of each disk was measured using the water displacement method^51^. To calculate wood specific gravity (WSG) of a sample we used the mean derived from the three disks (or pieces). Using the final fresh mass and water content we calculated the final dry mass, which enabled us to derive the mass loss from the initial dimensional measurements and initial WSG.

### Chemical analysis

We assessed initial chemical properties of the wood and bark. Disks samples stored at −20 °C were dried and ground to a powder for chemical analysis. Bark and wood were analysed separately. In addition, final chemical properties of remaining bark were also determined. The total carbon (C), nitrogen (N), phosphorus (P), potassium (K), water soluble sugar content, neutral detergent fiber (NDF), acid detergent fiber (ADF), and acid detergent lignin (ADL) were measured for both bark litter and wood. In addition, tannin content was measured for bark and fiber content was measured for wood. C and N were determined by Vario Max CN element analyzer (Elementa Analsensysteme, Germany). For P, and K measurements, samples were diluted with HCl and digested with HNO_3_-HClO_4_. These were measured with an inductively coupled plasma atomic emission spectrometer (iCAP 6300, IRIS Advantage, E R, Thermo Fisher Scientific, USA). Water-soluble sugars were analysed with Analyzer 3 (SEAL Analytical GmbH, Germany). Tannin content was measured with UV-visible spectrometer (UV 2450, SHIMADZU, Japan). NDF, ADF, ADL, fiber contents were determined using Fibertec ™ 2010 (FOSS Analytical AB, Sweden). ADL, NDF and ADF were later converted to lignin, hemicellulose and cellulose following equations in Chen *et al*.^52^.

### Data analysis

We assessed the decomposition of both bark litter and branches through the loss of dry mass over time. We modeled the percentage of mass loss of bark litter (logit transformed following^53^ over 12 months as function of number of days incubation, species, treatment (litter-bag type) and all two-way interactions. We modeled the percentage mass loss from wood branches over 24 months as function of number of days incubation, species and bark treatment (with/without bark) and all two-way interactions.

We used Akaike information criteria (AIC) to select optimal models, the function ANOVA to examine effect of each independent variable. All the analyses were performed in R version 3.3.1 (R Foundation for Statistical Computing^54^) and the nlme package^55^, using the modeling function *gls (generalized least squared*). Where differences among species were significant, we conducted post-hoc multiple comparison pairwise tests using the functions *cld* and *lsmeans* from the lsmeans package^56^. Model fit was investigated by plotting residuals against standardised effect sizes.

## Additional Information

**How to cite this article**: Dossa, G. G. O. *et al*. Factors controlling bark decomposition and its role in wood decomposition in five tropical tree species. *Sci. Rep.* **6**, 34153; doi: 10.1038/srep34153 (2016).

## Supplementary Material

Supplementary Information

## Figures and Tables

**Table 1:** Initial concentrations of chemical components in the bark and wood among the five tree species used in the decomposition experiment.

Species	Type of tissue	C (%)	N (%)	P (%)	K (%)	Sugar (%)	Lignin (%)	Hemicellulose (%)	Cellulose (%)	Tannins (%)	Fiber content (%)	Wood specific gravity
*Cunninghamia lanceolata*	Bark	49.7	0.703	0.087	0.377	1.03	42.9	6.36	20.17	1.40	NA	NA
	Wood	51.1	0.177	0.015	0.093	0.39	34.19	10.73	40.45	NA	69.32	0.805 (0.011)
*Tectona grandis*	Bark	44.1	0.418	0.021	0.131	3.99	20.47	21.26	36.52	0.44	NA	NA
	Wood	48.7	0.337	0.023	0.156	1.17	26.39	12.17	49	NA	65.66	0.649 (0.007)
*Dipterocarpus turbinatus*	Bark	45.3	0.491	0.016	0.146	1.03	33.76	10.38	35	2.21	NA	NA
	Wood	48.1	0.334	0.012	0.163	0.67	32.19	15.38	43.29	NA	69.56	0.850 (0.010)
*Toona ciliata*	Bark	45.0	0.857	0.096	0.473	3.90	22.36	11.88	30.18	7.26	NA	NA
	Wood	47.1	0.286	0.043	0.222	0.67	20.75	16.43	54.86	NA	67.2	0.698 (0.006)
*Kleinhovia hospita*	Bark	44.5	1.138	0.149	1.238	0.34	20.5	11.31	49.36	0.33	NA	NA
	Wood	47.6	0.62	0.106	0.758	0.78	34.1	10.67	44.99	NA	71.28	0.429 (0.004)

Values are laboratory duplicate averages; except for wood specific gravity which is the mean (±SE) of 24 samples.

**Table 2:** Model results for bark litter decomposition.

Variables	Numerator DF	F-values	P–value
Intercept	1	8.682	<0.0037
Number of days incubation	2	16.664	<0.0001
Litter-bag type	1	28.137	<0.0001
Species	4	3.334	<0.0120
Polynomial (number of days, 2): Species	8	3.239	0.002
Polynomial (number of days, 2): Litter bag type	2	27.520	<0.0001

Percentage mass loss (logit transformed) was modelled over 12 months incubation as function of number of days (2^nd^ order polynomial), litter-bag type (faunal access/exclusion), barks species and their interactive effects (for the full model and summary see Table S1 in supplementary material). There were five species: *Kleinhovia hospita, Tectona grandis, Cunninghamia lanceolata, Dipterocarpus turbinatus, Toona ciliata*. DF denotes degree of freedom. Denominator DF = 148.

**Table 3:** Model results for decomposition (mass loss) of branches.

Variables	Numerator DF	F-values	P–value
Intercept	1	0.402	0.527
Number of days incubation	2	13.053	<0.0001
Species	4	3.670	<0.008
Bark treatment	1	1.594	0.210
Species: Bark treatment	4	3.823	0.006
Polynomial (number of days, 2): Species	8	5.636	<0.0001

Percentage mass loss (logit transformed) was modelled over 24 months as function of number of days (2^nd^ order polynomial), bark treatment, species and all two-way interactions (for the full model and summary see Table S3 in supplementary material). There were five species: *Kleinhovia hospita, Tectona grandis, Cunninghamia lanceolata, Dipterocarpus turbinatus, Toona ciliata*, and bark treatment had two levels: branches with bark and branches without bark. DF denotes degree of freedom. Denominator DF = 98.
