# Supplementary material for: Factors controlling bark decomposition and its role in wood decomposition in five tropical tree species
Source: Sci Rep. 2016 Oct 4;6:34153. doi: 10.1038/srep34153 (PMC5048430; doi:10.1038/srep34153)
Supplement: Supplementary Information [file srep34153-s1.doc]

**Factors controlling bark decomposition and its role in wood decomposition in five tropical tree species.**

**Running title:** **Bark and wood decomposition**

Gbadamassi G. O. Dossa^1,2,3^, Ekananda Paudel^1,2,3^, Kunfang Cao^1,4^, Douglas Schaefer^1^, Rhett D. Harrison^2,5*^

^1^Key Laboratory of Tropical Forest Ecology, Xishuangbanna Tropical Botanical Garden, Chinese Academy of Sciences, Menglun, Mengla 666303, Yunnan, China

^2^Centre for Mountain Ecosystem Studies, Kunming Institute of Botany, Kunming 650201, Yunnan, China

^3^University of Chinese Academy of Sciences, Beijing 100039, China

^4^Ecophysiology and Evolution Group, State Key Laboratory for Conservation and Utilization of Subtropical Agro-Bioresources, and College of Forestry, Guangxi University, Nanning, Guangxi Province, 530004, China

^5^World Agroforestry Centre, East & Central Asia Regional Office, Kunming 650201, Yunnan, China.

**^*^**Corresponding author; e-mail: [r.harrison@cgiar.org](mailto:r.harrison@cgiar.org)

SUPPLEMENTARY MATERIALS

**Table S1**: **GLS model summary of percent mass loss (logit transformed) from bark litter after 12 mo incubation in a secondary rainforest at Xishuangbanna Tropical Botanical Garden.** Independent variables included number of days as polynomial second order (poly (Number_days, 2)), litter-bag type (Bag_type), species identity (Species), and the interaction between species and litter bag type. Species identity (Species) had 5 levels: *Kleinhovia hospita* (baseline), Species Tec_gra = *Tectona grandis*, Species Cun_lan= *Cunninghamia lanceolate,* Species Dip_tur *= Dipterocarpus turbinatus* and Species Too_cil= *Toona ciliata*. Litter-bag types (Bag_type) had two levels: Bag_typeCoarse_mesh = Coarse mesh (baseline, fauna access) and Bag_typeFine_mesh = Fine mesh (fauna exclusion)

|  | Value | Std.Error | t-value | p-value |
| --- | --- | --- | --- | --- |
| (Intercept) | -1.963 | 0.666 | -2.947 | 0.004 |
| poly(Number_days, 2)1 | 0.030 | 0.008 | 3.961 | 0.000 |
| poly(Number_days, 2)2 | 0.000 | 0.000 | -3.280 | 0.001 |
| Bag_typeFine_mesh | 1.776 | 0.335 | 5.304 | 0.000 |
| SpeciesTec_gra | -0.011 | 0.713 | -0.015 | 0.988 |
| SpeciesCun_lan | -1.416 | 0.677 | -2.092 | 0.038 |
| SpeciesDip_tur | -0.960 | 0.707 | -1.358 | 0.177 |
| SpeciesToo_cil | -1.232 | 0.691 | -1.782 | 0.077 |
| poly(Number_days, 2)1:SpeciesTec_gra | -0.011 | 0.008 | -1.405 | 0.162 |
| poly(Number_days, 2)2:SpeciesTec_gra | 0.000 | 0.000 | 1.600 | 0.112 |
| poly(Number_days, 2)1:SpeciesCun_lan | 0.002 | 0.008 | 0.288 | 0.773 |
| poly(Number_days, 2)2:SpeciesCun_lan | 0.000 | 0.000 | -0.218 | 0.828 |
| poly(Number_days, 2)1:SpeciesDip_tur | -0.004 | 0.008 | -0.449 | 0.654 |
| poly(Number_days, 2)2:SpeciesDip_tur | 0.000 | 0.000 | 0.795 | 0.428 |
| poly(Number_days, 2)1:SpeciesToo_cil | 0.002 | 0.008 | 0.284 | 0.777 |
| poly(Number_days, 2)2:SpeciesToo_cil | 0.000 | 0.000 | -0.369 | 0.713 |
| poly(Number_days, 2)1:Bag_typeFine_mesh | -0.024 | 0.004 | -6.040 | 0.000 |
| poly(Number_days, 2)2:Bag_typeFine_mesh | 0.000 | 0.000 | 5.303 | 0.000 |

**Table S2**: **Results of post-hoc pairwise Tukey tests between species for bark litter decomposition.** The values are on logit transformed scale with epsilon = 0.177, otherwise specified. Within the same level of bark treatment, species having the same group letter or number are not significant different from one another.

| Species | Litter bag type | Lsmean | Standard error | D.F. | Lower CL | Upper CL | Group | Lsmean (%) |
| --- | --- | --- | --- | --- | --- | --- | --- | --- |
| *Cunninghamia lanceolata* | Coarse mesh | 0.369 | 0.084 | 148 | 0.204 | 0.535 | 1 | 41.43 |
| *Toona ciliata* | Coarse mesh | 0.388 | 0.085 | 148 | 0.220 | 0.556 | 1 | 41.88 |
| *Dipterocarpus turbinatus* | Coarse mesh | 0.615 | 0.101 | 148 | 0.416 | 0.815 | 12 | 47.22 |
| *Tectona grandis* | Coarse mesh | 0.700 | 0.101 | 148 | 0.501 | 0.900 | 2 | 49.13 |
| *Kleinhovia hospita* | Coarse mesh | 1.509 | 0.143 | 148 | 1.226 | 1.792 | 3 | 64.19 |
| *Cunninghamia lanceolata* | Fine mesh | -0.391 | 0.063 | 148 | -0.515 | -0.267 | a | 22.64 |
| *Toona ciliata* | Fine mesh | -0.372 | 0.067 | 148 | -0.505 | -0.240 | a | 23.10 |
| *Dipterocarpus turbinatus* | Fine mesh | -0.145 | 0.086 | 148 | -0.315 | 0.025 | ab | 28.68 |
| *Tectona grandis* | Fine mesh | -0.060 | 0.086 | 148 | -0.231 | 0.111 | b | 30.79 |
| *Kleinhovia hospita* | Fine mesh | 0.748 | 0.132 | 148 | 0.488 | 1.009 | c | 50.18 |

**Table S3**: **GLS model summary for percent mass loss (logit transformed) from branches after 24 mo incubation in a secondary rainforest at Xishuangbanna Tropical Botanical Garden.** Independent variables included number of days (Number_days), bark treatment (bark.trtment), species identity (Species), and the interaction between species and bark treatment. Species identity (Species) had 5 levels: *Kleinhovia hospita* (baseline), Species Tec_gra = *Tectona grandis*, Species Cun_lan= *Cunninghamia lanceolate, Species Dip_tur = Dipterocarpus turbinatus* and Species Too_cil= *Toona ciliata*. Bark treatment (bark.trtment) had two levels: bark.trtment_bark = log with bark (baseline), and bark.trtmentno_bark = log without bark

|  | Value | Std.Error | t-value | p-value |
| --- | --- | --- | --- | --- |
| (Intercept) | -0.425 | 0.670 | -0.634 | 0.527 |
| poly(Number_days, 2)1 | 0.013 | 0.004 | 3.434 | 0.001 |
| poly(Number_days, 2)2 | 0.000 | 0.000 | -2.612 | 0.010 |
| SpeciesTec_gra | -1.620 | 0.976 | -1.660 | 0.100 |
| SpeciesCun_lan | -0.893 | 0.985 | -0.907 | 0.367 |
| SpeciesDip_tur | -3.425 | 1.029 | -3.328 | 0.001 |
| SpeciesToo_cil | -2.729 | 0.971 | -2.810 | 0.006 |
| bark.trtmentno_bark | -0.412 | 0.326 | -1.263 | 0.210 |
| SpeciesTec_gra:bark.trtmentno_bark | -1.048 | 0.454 | -2.309 | 0.023 |
| SpeciesCun_lan:bark.trtmentno_bark | -0.473 | 0.457 | -1.035 | 0.303 |
| SpeciesDip_tur:bark.trtmentno_bark | 0.640 | 0.455 | 1.406 | 0.163 |
| SpeciesToo_cil:bark.trtmentno_bark | -0.213 | 0.455 | -0.468 | 0.641 |
| poly(Number_days, 2)1:SpeciesTec_gra | -0.007 | 0.005 | -1.375 | 0.172 |
| poly(Number_days, 2)2:SpeciesTec_gra | 0.000 | 0.000 | 1.320 | 0.190 |
| poly(Number_days, 2)1:SpeciesCun_lan | -0.012 | 0.005 | -2.208 | 0.030 |
| poly(Number_days, 2)2:SpeciesCun_lan | 0.000 | 0.000 | 2.192 | 0.031 |
| poly(Number_days, 2)1:SpeciesDip_tur | 0.001 | 0.005 | 0.179 | 0.858 |
| poly(Number_days, 2)2:SpeciesDip_tur | 0.000 | 0.000 | 0.754 | 0.453 |
| poly(Number_days, 2)1:SpeciesToo_cil | -0.001 | 0.005 | -0.170 | 0.866 |
| poly(Number_days, 2)2:SpeciesToo_cil | 0.000 | 0.000 | 0.711 | 0.479 |

**Table S4**: **Results of post-hoc pairwise Tukey tests between species for wood decomposition.** The values are on logit transformed scale with epsilon = 0.0603, otherwise specified. Within the same level of bark treatment, species having the same group letter or number are not significant different from one another.

| Species | Bark treatment | Lsmean | Standard error | D.F. | Lower CL | Upper CL | Group | Lsmean (%) |
| --- | --- | --- | --- | --- | --- | --- | --- | --- |
| *Tectona grandis* | Bark | -0.421 | 0.177 | 98 | -0.773 | -0.069 | 1 | 33.60 |
| *Cunninghamia lanceolata* | Bark | -0.413 | 0.186 | 98 | -0.782 | -0.045 | 1 | 33.78 |
| *Toona ciliata* | Bark | 0.389 | 0.177 | 98 | 0.038 | 0.741 | 2 | 53.58 |
| *Dipterocarpus turbinatus* | Bark | 0.570 | 0.180 | 98 | 0.212 | 0.928 | 2 | 57.84 |
| *Kleinhovia hospita* | Bark | 2.489 | 0.181 | 98 | 2.130 | 2.847 | 3 | 86.30 |
| *Tectona grandis* | no_bark | -1.881 | 0.261 | 98 | -2.399 | -1.362 | a | 07.20 |
| *Cunninghamia lanceolata* | no_bark | -1.298 | 0.261 | 98 | -1.817 | -0.780 | a | 15.42 |
| *Toona ciliata* | no_bark | -0.235 | 0.263 | 98 | -0.756 | 0.287 | b | 38.13 |
| *Dipterocarpus turbinatus* | no_bark | 0.798 | 0.261 | 98 | 0.280 | 1.317 | c | 62.93 |
| *Kleinhovia hospita* | no_bark | 2.077 | 0.274 | 98 | 1.533 | 2.621 | d | 82.83 |


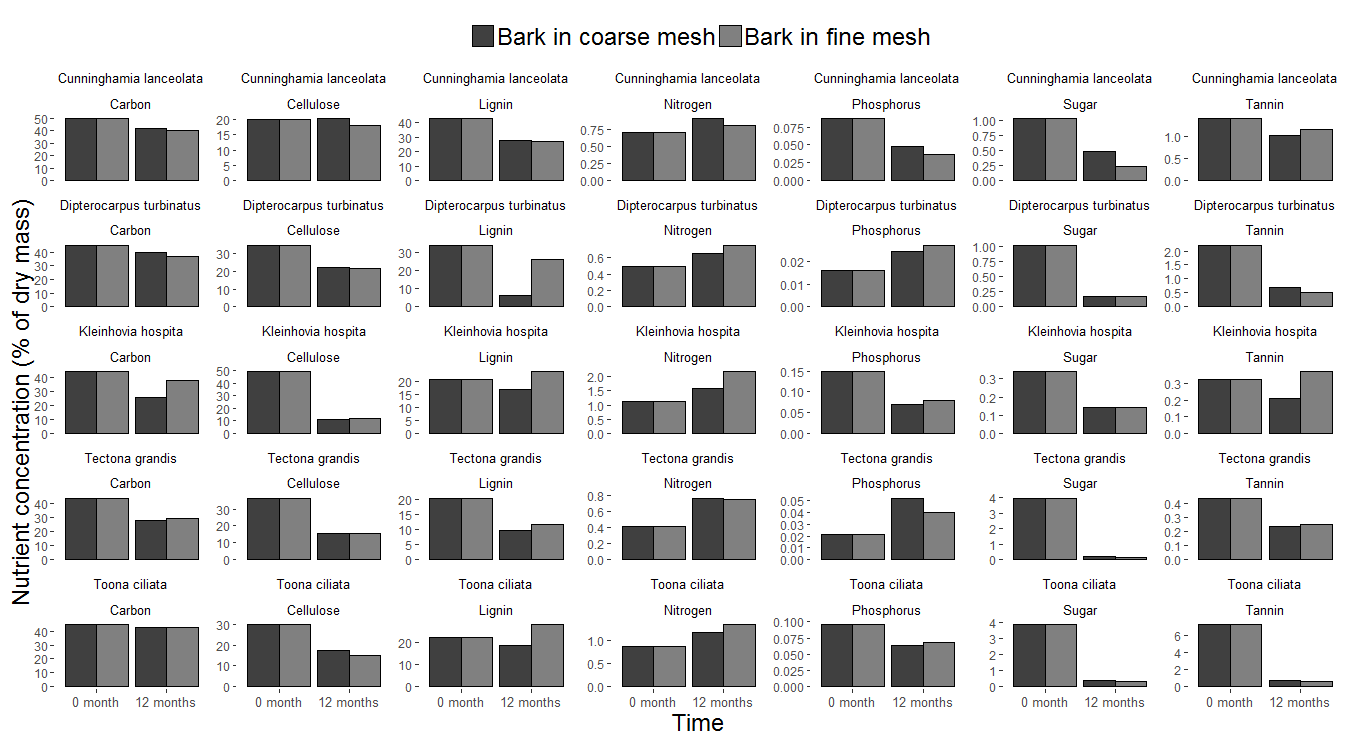


**Figure S1**: Changes in bark litter chemistry from the beginning (0 month of incubation) to the end (12 months of incubation) of a decomposition experiment a secondary rain forest at Xishuangbanna Tropical Botanical Garden for five tree species (*Kleinhovia hospita, Tectona grandis, Cunninghamia lanceolata, Dipterocarpus turbinatus,* and *Toona ciliata*) with respect to faunal exclusion (dark black bars, fine mesh, mesh size = 0.068 mm) and faunal access (grey bars, coarse mesh, mesh size = 4.75 mm upper side). Samples were mixed prior to chemical analysis. Values are laboratory duplicate averages. For the analysis of the final concentrations, due to the amount of remaining bark, only two samples (n=2) per each bag type and species were sufficient for analysis.


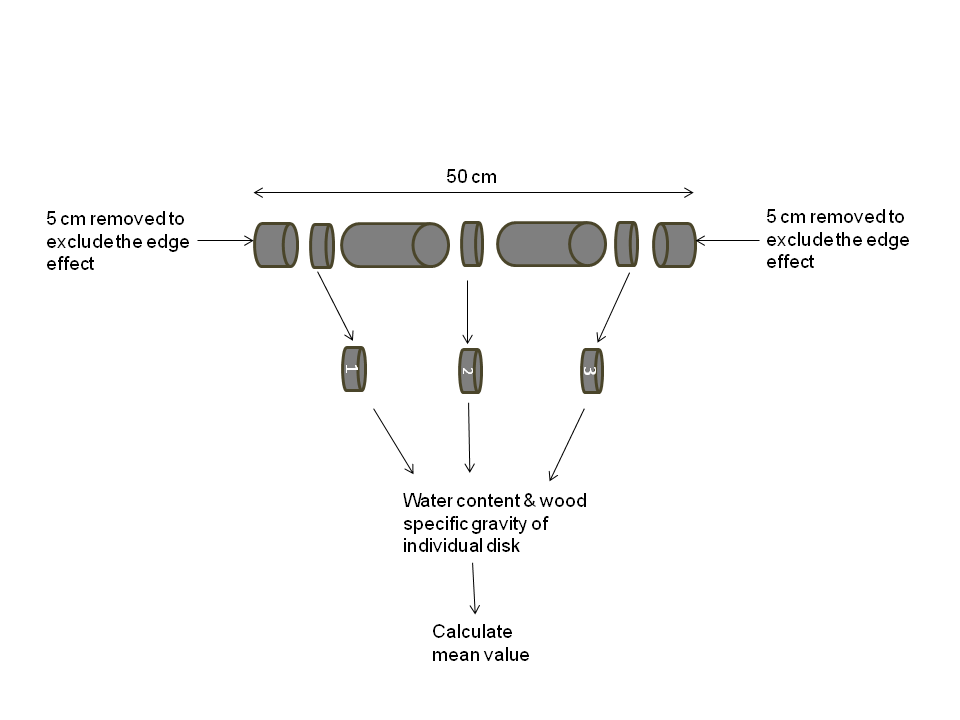


**Figure S2**: **Schematic diagram of sampling discs from an individual harvested branch**
